# Effect of Broad‐Spectrum Antibiotic Prophylaxis on Post‐Pancreatoduodenectomy Infectious Complications: Nationwide Inpatient Database Study in Japan

**DOI:** 10.1002/ags3.70198

**Published:** 2026-02-26

**Authors:** Hiroki Kitagawa, Shotaro Aso, Yuki Hirano, Kenichiro Uemura, Keita Kouzu, Satoru Matsuda, Hiroki Matsui, Kiyohide Fushimi, Junichi Sasaki, Yuko Kitagawa, Hideo Yasunaga

**Affiliations:** ^1^ Department of Infectious Diseases Hiroshima University Hospital Hiroshima Japan; ^2^ Department of Surgery, Graduate School of Biomedical and Health Sciences Hiroshima University Hiroshima Japan; ^3^ Department of Health Services Research, Graduate School of Medicine The University of Tokyo Tokyo Japan; ^4^ Department of Hepatobiliary‐Pancreatic and Gastrointestinal Surgery International University of Health and Welfare School of Medicine Chiba Japan; ^5^ Department of Surgery National Defense Medical College Saitama Japan; ^6^ Department of Surgery Keio University School of Medicine Tokyo Japan; ^7^ Department of Clinical Epidemiology and Health Economics, School of Public Health The University of Tokyo Tokyo Japan; ^8^ Department of Health Policy and Informatics Institute of Science Tokyo Graduate School Tokyo Japan; ^9^ Department of Emergency and Critical Care Medicine Keio University School of Medicine Tokyo Japan

**Keywords:** antibiotics, cephalosporins, pancreatoduodenectomy, piperacillin‐tazobactam, surgical site infection

## Abstract

**Background:**

Despite improvements in surgical techniques and perioperative management, the incidence of postoperative surgical site infection after pancreatoduodenectomy remains high. This study aim to assess whether broad‐spectrum antibiotic prophylaxis can reduce complications after pancreatoduodenectomy compared with standard care antibiotics.

**Methods:**

Data of patients who underwent pancreatoduodenectomy between July 2010 and March 2022 were extracted from a nationwide Japanese inpatient database. First‐ and second‐generation cephalosporins were designated as narrow‐spectrum, and third‐ and fourth‐generation cephalosporins and piperacillin–tazobactam as broad‐spectrum antibiotics. Patients who received either narrow‐ or broad‐spectrum antibiotics on the day of surgery were included in the analysis. Using propensity‐score stabilized inverse probability of treatment weighting, the postoperative complications were compared between patients who received antimicrobial prophylaxis with narrow‐spectrum antibiotics versus broad‐spectrum antibiotics.

**Results:**

From among 45 099 eligible patients, 36 742 (81.5%) and 8357 (18.5%) patients received narrow‐ and broad‐spectrum antibiotics, respectively. After stabilized inverse probability of treatment weighting, the use of broad‐spectrum antibiotics bore a significant association with the reduction in intra‐abdominal infections [risk difference (RD), −7.4; 95% confidence interval (CI), −8.7 to −6.0], postoperative pancreatic fistula (RD, −3.0; 95% CI, −4.0 to −1.9), post‐pancreatectomy hemorrhage (RD, −1.5; 95% CI, −1.9 to −1.1). The use of broad‐spectrum antibiotics was also associated with a shorter postoperative length of hospital stay and lower total hospitalization costs. The proportion of *Clostridioides difficile* infection did not differ between the groups.

**Conclusions:**

The administration of broad‐spectrum antibiotics as antimicrobial prophylaxis was associated with better in‐hospital postoperative outcomes compared with narrow‐spectrum in patients undergoing pancreatoduodenectomy.

## Graphical abstract

Using a nationwide Japanese inpatient database, we evaluated whether broad‐spectrum antibiotic prophylaxis improves postoperative outcomes after pancreatoduodenectomy compared with narrow‐spectrum antibiotics. In propensity score–weighted analyses of 45 099 patients, broad‐spectrum prophylaxis was associated with significantly lower rates of intra‐abdominal infection, postoperative pancreatic fistula, and post‐pancreatectomy hemorrhage, as well as shorter hospital stay and lower costs, without an increased risk of *Clostridioides difficile* infection. These findings suggest that broad‐spectrum antibiotic prophylaxis may be associated with improved in‐hospital outcomes after pancreatoduodenectomy.

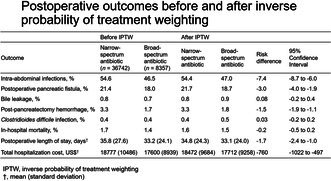

## 1 Introduction

Pancreatoduodenectomy (PD) is a common surgical procedure for both benign and malignant diseases of the periampullary region. Despite the dramatic improvements in the surgical techniques for PD, postoperative morbidity has persistently remained high [1, 2]. Surgical site infections (SSIs) and postoperative pancreatic fistula (POPF) are the most common complications after PD [1, 2]. These complications can have a deleterious effect on the quality of life, delay or prevent the administration of adjuvant therapies, and are associated with a lower overall survival [3, 4].

The effect of broad‐spectrum antibiotic prophylaxis on the infectious complications after PD remains unknown. The current guidelines recommend first‐ or second‐generation cephalosporins as perioperative prophylaxis for PD [5, 6]. Previous studies have shown that broad‐spectrum prophylaxis using third‐ and fourth‐generation cephalosporins and piperacillin‐tazobactam reduced the incidence of SSI and POPF after PD compared with standard antibiotic prophylaxis [7, 8]. Recently, a randomized controlled trial showed that perioperative antimicrobial prophylaxis using piperacillin‐tazobactam reduced the risk of postoperative SSI and POPF compared with cefoxitin in patients undergoing PD [9]. However, in the previous study, piperacillin–tazobactam was not associated with a reduction in SSI incidence in patients without biliary stents [9]. To date, no large‐scale study utilizing real‐world data that encompasses diverse patient and hospital populations has been conducted to generate more generalizable evidence on the effectiveness of peri‐operative broad‐spectrum antimicrobial prophylaxis in PD. Therefore, whether routine broad‐spectrum agents such as piperacillin–tazobactam should be used for antibiotic prophylaxis in PD has not been fully investigated.

In the present study, we aimed to compare the in‐hospital outcomes, including intra‐abdominal infectious after PD, between patients who received antimicrobial prophylaxis with narrow‐spectrum antibiotics versus broad‐spectrum antibiotics, using data extracted from a nationwide inpatient database from Japan.

## 2 Methods

### 2.1 Data Source

This retrospective cohort study utilized data from the Diagnosis Procedure Combination database, a nationwide repository of inpatient information maintained in Japan [10]. This database collects approximately 7 million discharge summaries and hospital administrative claims data from approximately 1800 health care facilities annually. Participation in the database is mandatory for all university hospitals, whereas other hospitals participate voluntarily. The database covers approximately 90% of all tertiary care emergency hospitals and 44% of institutions certified by the Japanese Surgical Society. The requirement for informed consent was waived because of the anonymous nature of the data. This study was approved by the Institutional Review Board of the University of Tokyo [approval number: 3501‐(5)].

The Diagnosis Procedure Combination database includes information on the following variables: sex; age; body mass index; smoking index; diagnosis and comorbidities on admission and complications after admission, recorded using the International Classification of Diseases, Tenth Revision codes (ICD‐10); medication(s) administered during hospitalization; interventional/surgical procedures recorded according to the original Japanese codes; unique hospital identifier; discharge status; and hospitalization cost. The discharge summary for each patient is coded by the attending physicians. Previous validation studies have shown that this database possesses highly accurate data on the primary diagnosis, surgical procedures, comorbidities, and postoperative complications [11, 12, 13, 14].

### 2.2 Study Protocol

The data of patients who underwent PD between July 2010 and March 2022 and received narrow‐ or broad‐spectrum antibiotics on the day of surgery were extracted from the database. Patients who underwent hepatopancreatoduodenectomy were excluded. Narrow‐spectrum antibiotics were defined as first‐generation cephalosporins and second‐generation cephalosporins as well as cephamycins such as cefmetazole and flomoxef. Third‐ and fourth‐generation cephalosporins and piperacillin‐tazobactam were designated as broad‐spectrum antibiotics. The exclusion criteria were as follows: age below 18 years, patients who did not receive any antibiotics or received non‐target antibiotics on the day of surgery, those who underwent PD more than 7 days after admission, receipt of any antibiotics before surgery, duration of general anesthesia < 180 min, or missing information on the fiscal year of PD. We divided patients into two groups: [1] those who received narrow‐spectrum antibiotics on the day of surgery (narrow‐spectrum antibiotic group) and [2] those who received broad‐spectrum antibiotics on the day of surgery (broad‐spectrum antibiotics group). Patients who received both narrow‐ and broad‐spectrum antibiotics on the day of surgery were assigned to the broad‐spectrum antibiotics group.

The primary outcome was intra‐abdominal infection, defined as the occurrence of anastomotic leakage, POPF, bile leakage, or intra‐abdominal abscess. The secondary outcomes included POPF, bile leakage, post‐pancreatectomy hemorrhage, *Clostridioides difficile* infection, in‐hospital mortality, postoperative length of stay, and total hospitalization cost in USD. We converted the costs from Japanese yen using an exchange rate of 146.6 JPY/USD, as of June 22, 2025. The ICD‐10 and procedure codes used to define these postoperative complications are shown in Table S1.

### 2.3 Statistical Analysis

To adjust for the differences in the baseline characteristics between the two groups, stabilized inverse probability of treatment weighting (IPTW) analyses were conducted using propensity scores [15]. Stabilized IPTW uses propensity scores and adjusts for measured potential confounders while preserving the sample size. The propensity score was estimated using a multivariate logistic regression model based on the patient‐related background factors (sex, age, body mass index, smoking episode, Barthel index, Charlson Comorbidity Index, hypertension, diabetes, chronic obstructive pulmonary disease, renal failure, pathological diagnosis, and fiscal year), treatment factors [preoperative biliary stent, procedure (open surgery or minimally invasive surgery), duration of anesthesia, and transfusion on the day of surgery], and hospital factors (hospital type and hospital volume). Comorbidities were scored using the Charlson Comorbidity Index according to the protocol for the ICD‐10 codes [16] and classified into four groups: 0, 1, 2, and ≥ 3. The Charlson Comorbidity Index is calculated exclusively based on comorbid conditions, with the primary disease being excluded from the scoring process. The type of hospital was categorized as a teaching or non‐teaching institution. Hospital volume was defined as the number of PDs performed annually in each hospital and categorized into tertiles: 1–6, 7–13, and > 13 cases annually.

The propensity‐score inverse probability of treatment weights were stabilized by multiplication with the overall proportion of each group. Absolute standardized differences were calculated to examine the balance in the baseline covariates between the two groups before and after adjustment. An absolute standardized difference of < 0.10 indicated well‐balanced covariates [17].

We performed subgroup analyses for the primary outcome using IPTW stratified by the receipt of a preoperative bile stent and the presence of pancreatic adenocarcinoma.

The *t*‐test was used to compare continuous variables, and the chi‐square test was used to compare the categorical variables between the two groups. Statistical significance was set at *p* < 0.05. All statistical analyses were conducted using STATA version 19.5 (StataCorp LLC, College Station, TX).

## 3 Results

**FIGURE 1:**
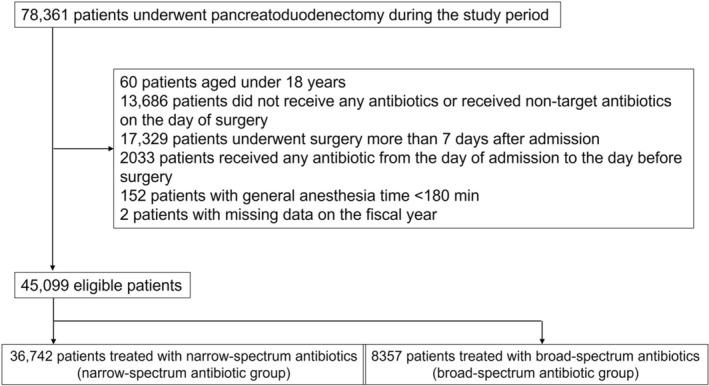
Flow chart for identifying patients eligible for this study.

Figure 1 presents a flowchart illustrating the identification of eligible patients. During the study period, 78 361 patients underwent PD. Of these, 45 099 patients were eligible for enrollment in this study. These patients were divided into the narrow‐ (*n* = 36 742) and broad‐spectrum antibiotic (*n* = 8357) groups. In the broad‐spectrum antibiotic group, 7529 (90.1%) patients received third‐ or fourth‐generation cephalosporins, while 828 (9.9%) patients received piperacillin–tazobactam. Table 1 shows the participants' baseline characteristics. Before IPTW, patients in the broad‐spectrum antibiotic group were more likely to have biliary tract cancer, receive open surgery, receive red blood cell transfusion on the day of surgery, be treated at high‐volume hospitals compared with patients in the narrow‐spectrum antibiotic group. The fiscal year of surgery differed between the two groups. After IPWT, all covariates were well‐balanced between the two groups.

**TABLE 1:** Patients' background characteristics.

Variable	Before IPTW		ASD	After IPTW		ASD
	Narrow‐spectrum antibiotic (*n* = 36 742)	Broad‐spectrum antibiotic (*n* = 8357)		Narrow‐spectrum antibiotic	Broad‐spectrum antibiotic	
Male	19 886 (59.9)	4865 (62.0)	0.036	(59.3)	(59.4)	0.001
Age (year), mean (SD)	69.1 (10.3)	69.6 (10.1)	0.050	69.1 (10.3)	69.1 (10.3)	0.003
Body mass index (kg/m^2^)						0.006
< 18.5	4130 (11.3)	894 (10.7)	0.059	(11.1)	(11.2)	
18.5–24.9	25 128 (68.7)	5908 (70.9)		(69.0)	(68.9)	
25.0–29.9	6473 (17.7)	1385 (16.6)		(17.6)	(17.5)	
≥ 30.0	865 (2.4)	146 (1.8)		(2.3)	(2.3)	
Smoking episode	14 448 (43.2)	3273 (43.8)	0.011	(43.4)	(43.5)	0.002
Barthel index < 95	1034 (2.9)	293 (3.5)	0.039	(2.9)	(2.9)	0.003
Charlson Comorbidity Index						0.009
0	26 397 (71.8)	5852 (70.0)	0.044	(72.1)	(72.3)	
1	2189 (6.0)	532 (6.4)		(5.7)	(5.7)	
2	6014 (16.4)	1486 (17.8)		(16.3)	(16.3)	
≥ 3	2142 (5.8)	487 (5.8)		(5.8)	(5.7)	
Comorbidities						
Hypertension	10 231 (27.8)	2246 (26.9)	0.022	(27.6)	(27.6)	< 0.001
Diabetes mellitus	11 805 (32.1)	2680 (32.1)	0.001	(31.0)	(30.7)	0.006
Chronic obstructive pulmonary disease	614 (1.7)	122 (1.5)	0.017	(1.7)	(1.6)	0.002
Renal failure	538 (1.5)	129 (1.5)	0.007	(1.4)	(1.4)	0.001
Pathology						
Pancreatic adenocarcinoma	12 609 (34.3)	2874 (34.4)	0.002	(34.1)	(34.1)	0.001
Biliary tract cancer	8306 (22.6)	2308 (27.6)	0.12	(23.8)	(23.9)	0.003
Duodenal adenocarcinoma	6588 (17.9)	1493 (17.9)	0.002	(17.8)	(17.9)	0.003
IPMN	1967 (5.4)	401 (4.8)	0.025	(5.3)	(5.3)	< 0.001
Pancreatic neuroendocrine tumor	553 (1.5)	67 (0.8)	0.066	(1.4)	(1.4)	0.003
Duodenal neuroendocrine tumor	463 (1.3)	59 (0.7)	0.056	(1.2)	(1.2)	0.003
Chronic pancreatitis	242 (0.7)	79 (0.9)	0.032	(0.7)	(0.7)	0.002
Preoperative biliary stent	12 379 (33.7)	3470 (41.5)	0.16	(36.2)	(36.6)	0.009
Open surgery	35 697 (97.2)	8261 (98.9)	0.11	(97.3)	(97.4)	0.002
Duration of anesthesia (min), mean (SD)	527.4 (379.1)	510.3 (127.3)	0.072	522.2 (379.4)	522.2 (131.8)	< 0.001
Transfusion on the day of surgery						
Red blood cell	5997 (16.3)	1742 (20.8)	0.12	(16.7)	(17.1)	0.010
Fresh‐frozen plasma	2884 (7.7)	817 (9.8)	0.072	(7.9)	(8.1)	0.008
Platelet concentrate	215 (0.6)	68 (0.8)	0.027	(0.6)	(0.6)	0.005
Teaching hospital	20 762 (56.5)	4635 (55.5)	0.021	(56.2)	(55.8)	0.019
Hospital volume (cases/year)						
1–6	11 459 (34.5)	2191 (28.6)	0.15	(34.3)	(35.2)	0.019
7–13	10 698 (32.2)	2922 (38.2)		(32.7)	(32.4)	
> 13	11 060 (33.3)	2546 (33.2)		(33.0)	(32.4)	
Fiscal year						
2010	1117 (3.0)	212 (2.5)	0.10	(2.7)	(2.8)	0.019
2011	1665 (4.5)	352 (4.2)		(4.2)	(4.3)	
2012	2106 (5.7)	465 (5.6)		(5.1)	(5.3)	
2013	2243 (6.1)	546 (6.5)		(5.7)	(5.8)	
2014	2733 (7.4)	662 (7.9)		(7.3)	(7.3)	
2015	2677 (7.3)	790 (9.5)		(7.7)	(7.7)	
2016	3140 (8.5)	721 (8.6)		(8.7)	(8.7)	
2017	3265 (8.9)	759 (9.1)		(9.0)	(8.9)	
2018	3513 (9.6)	713 (8.5)		(9.5)	(9.3)	
2019	3307 (9.0)	801 (9.6)		(9.2)	(9.5)	
2020	3708 (10.1)	810 (9.7)		(10.5)	(10.4)	
2021	3638 (9.9)	790 (9.5)		(10.3)	(10.0)	
2022	3630 (9.9)	736 (8.8)		(10.1)	(10.0)	

*Note:* Values are *n* (%) unless otherwise indicated.

Abbreviations: ASD, absolute standardized difference; IPMN, intraductal papillary mucinous neoplasm; IPTW, inverse probability of treatment weighting; SD, standard deviation.

Table 2 shows the primary and secondary outcomes before and after IPTW. After IPTW, the incidence of intra‐abdominal infection [47.0% vs. 54.4%; risk difference (RD), −7.4; 95% confidence interval (CI), −8.7 to −6.0], POPF (18.7% vs. 21.7%; RD, −3.0; 95% CI, −4.0 to −1.9), and post‐pancreatectomy hemorrhage (1.8% vs. 3.3%; RD, −1.5; 95% CI, −1.9 to −1.1) was significantly lower in the broad‐spectrum antibiotic group than in the narrow‐spectrum antibiotic group. The use of broad‐spectrum antibiotics was associated with a shorter postoperative length of hospital stay (Difference, −1.7 days; 95% CI, −2.4 to −1.0) and lower total hospitalization costs (Difference, US$ −760; 95% CI, −1022 to −497) compared with the narrow‐spectrum antibiotic group. The proportions of *C. difficile* infection (RD, −0.03; 95% CI, −0.2 to 0.2) and in‐hospital mortality (RD, −0.2; 95% CI, −0.5 to 0.2) did not differ between the two groups.

**TABLE 2:** Postoperative outcomes before and after inverse probability of treatment weighting.

Outcome	Before IPTW		After IPTW			
	Narrow‐spectrum antibiotic (*n* = 36 742)	Broad‐spectrum antibiotic (*n* = 8357)	Narrow‐spectrum antibiotic	Broad‐spectrum antibiotic	Risk difference	95% Confidence interval
Intra‐abdominal infections, %	54.6	46.5	54.4	47.0	−7.4	−8.7 to −6.0
Postoperative pancreatic fistula, %	21.4	18.0	21.7	18.7	−3.0	−4.0 to −1.9
Bile leakage, %	0.8	0.7	0.8	0.9	0.08	−0.2 to 0.4
Post‐pancreatectomy hemorrhage, %	3.3	1.7	3.3	1.8	−1.5	−1.9 to −1.1
*Clostridioides difficile* infection, %	0.4	0.4	0.4	0.5	0.03	−0.2 to 0.2
In‐hospital mortality, %	1.7	1.4	1.6	1.5	−0.2	−0.5 to 0.2
Postoperative length of stay, days^a^	35.8 (27.6)	33.2 (24.1)	34.8 (24.3)	33.1 (24.0)	−1.7	−2.4 to −1.0
Total hospitalization cost, US$^a^	18 777 (10486)	17 600 (8939)	18 472 (9684)	17 712 (9258)	−760	−1022 to −497

Abbreviation: IPTW, inverse probability of treatment weighting.

^a^ Mean (standard deviation).

Table 3 shows the results of the subgroup analyses for intra‐abdominal infection. The use of broad‐spectrum antibiotics was associated with a lower incidence of intra‐abdominal infections in patients with and those without a preoperative biliary stent.

**TABLE 3:** Subgroup analysis of intra‐abdominal infections after inverse probability of treatment weighting.

Subgroup	Risk difference (95% confidence interval)	*p*
Preoperative bile stent		
Yes	−9.1 (−11.2 to −7.0)	< 0.001
No	−6.4 (−8.2 to −4.6)	< 0.001
Pancreatic adenocarcinoma		
Yes	−6.9 (−9.2 to −5.0)	< 0.001
No	−7.6 (−9.4 to −6.0)	< 0.001

## 4 Discussion

This nationwide database study aimed to assess the effect of antimicrobial prophylaxis with broad‐spectrum antibiotics versus narrow‐spectrum antibiotics on the in‐hospital outcomes after PD. Our findings showed that antimicrobial prophylaxis with broad‐spectrum antibiotics was associated with a lower incidence of intra‐abdominal infection compared with narrow‐spectrum antibiotics. Moreover, broad‐spectrum antibiotics use was associated with a lower incidence of POPF and post‐pancreatectomy hemorrhage, shorter postoperative length of stay, and lesser total hospitalization costs. To the best of our knowledge, this is the first study to analyze the association between the choice of agent for antimicrobial prophylaxis and in‐hospital outcomes after PD using a large‐scale database. In addition, our study provided complementary evidence of the effectiveness of broad‐spectrum prophylaxis across diverse patient groups and institutional settings. These findings could expand the external validity of prior trial data and underscore the practical influence of antibiotic selection in routine clinical care.

Our results were consistent with those of previous studies suggesting that the use of third‐ or fourth‐generation cephalosporins and piperacillin–tazobactam as perioperative prophylaxis in PD curtailed the incidence of postoperative SSIs [7, 8, 9]. A recent randomized controlled trial showed that the use of piperacillin–tazobactam as perioperative prophylaxis yielded a lower incidence of postoperative SSI and POPF than that with cefoxitin in patients undergoing PD [9]. In a previous study, the presence of cefoxitin‐resistant organisms on intraoperative bile culture, 93% of which contained either *Enterobacter* or *Enterococcus* spp., was associated with the development of SSI and POPF in participants treated with cefoxitin but not in those treated with piperacillin–tazobactam [18]. Other studies also reported that *Enterobacter* and *Enterococcus* spp. were the most prevalent microbiological isolates in bile culture and causative microbes of SSI in patients who underwent PD, especially patients with preoperative biliary drainage [7, 19].

In contrast to a recent randomized controlled trial that focused on piperacillin–tazobactam [9], in the present study, the category of broad‐spectrum antibiotics comprised agents with heterogeneous antimicrobial spectra, including third‐ and fourth‐generation cephalosporins and piperacillin–tazobactam. Previous studies have suggested that piperacillin–tazobactam has activity against *Enterococcus* species, especially *Enterococcus faecalis* , while *Enterococcus* species are intrinsically resistant to cephalosporins; thus, the administration of piperacillin–tazobactam may be associated with a significant reduction in the incidence of SSI and POPF after PD [8, 9]. Third‐ or fourth‐generation cephalosporins and piperacillin–tazobactam have activity against *Enterobacter cloacae* , *Citrobacter freundii* , and *Klebsiella aerogenes* , while these organisms are intrinsically resistant to first‐ and second‐generation cephalosporins. Some previous studies have shown that third‐ or fourth‐generation cephalosporins reduced the incidence of SSI compared with standard antibiotic prophylaxis [7]. However, a retrospective study showed that antibiotic prophylaxis using piperacillin–tazobactam in patients who underwent pancreaticoduodenectomy significantly reduced organ/space SSI compared with ceftriaxone [20]. The optimal antimicrobial prophylaxis for PD may vary according to the preoperative bile culture results and local antimicrobial susceptibility patterns.

The risk of infection may also be related to preoperative biliary drainage, which introduces bacteria into the normally sterile biliary tree and is associated with high SSI rates [21, 22, 23]. Subgroup analysis conducted in a previous randomized controlled trial revealed that the use of piperacillin–tazobactam was associated with a lower incidence of SSI among patients with biliary stents. This association was not observed in patients without biliary stents, possibly because the statistical power was constrained by the small sample size [9]. However, in this study, subgroup analysis showed the use of broad‐spectrum antibiotics was associated with a lower proportion of postoperative intra‐abdominal infections in patients with and those without a preoperative biliary stent. Therefore, broad‐spectrum antibiotics may be recommended as the standard prophylactic regimen for PD, irrespective of the presence or absence of a preoperative biliary stent.

Broad‐spectrum antibiotics are associated with an elevated risk of *C. difficile* infection and acquisition of multi‐drug resistant organisms [24, 25]. In this study, there was no difference in the proportion of *C. difficile* infection between the narrow‐ and broad‐spectrum antibiotic groups. In addition, the overall incidence of CDI was comparable to that reported in a previous randomized controlled trial, which ranged between 0.3% and 3.5% [9]. Therefore, based on the efficacy, safety, and cost, our findings support the use of broad‐spectrum antibiotics including third and fourth‐generation cephalosporins, piperacillin–tazobactam for antimicrobial prophylaxis of PD.

This study has some limitations. First, we could not obtain detailed data on clinical variables such as neoadjuvant chemotherapy and radiation, pancreatic duct size, pancreatic gland texture, vascular resection, and severity of intra‐abdominal infections, such as POPF and post‐pancreatectomy hemorrhage [26, 27]. Second, the Diagnosis Procedure Combination database does not contain information on the timing of antimicrobial prophylaxis. The Japanese SSI guidelines recommend initiation of antimicrobial administration within 60 min before the surgical incision and redosing during surgery in cases of prolonged procedures when the procedural duration exceeds the half‐life of the prophylactic antimicrobial agent [28], in line with other guidelines [5, 6]. We consider adherence to these recommendations for PD to be high in Japan, analogous to the high adherence during gastrectomy (> 90%) reported by a national surveillance study [29]. Third, because the dataset was not intended for surveillance purposes, minor wound infection (Clavien–Dindo grade I) may have been undetected. The proportion of postoperative intra‐abdominal infections in the current study was 45%–55%, which was relatively higher than that reported by previous studies [2, 7, 8, 9]. A national surveillance study of SSI of PD (2012–2023) reported that the proportion of SSI was approximately 25% [30]. This discrepancy may be related to the real‐world origin of the dataset, which includes a wide range of hospitals beyond high‐volume centers. Fourth, minimally invasive PD without and with lymph node dissection has been reimbursed separately from open PD under the Japanese public universal insurance system since April 2016 and April 2020, respectively. Hence, information on the minimally invasive PD before April 2016 is not available. Fifth, since distinguishing whether antibiotics administered beyond postoperative day 1 were given for prophylactic purposes or therapeutic management of emerging complications was impossible, the duration of prophylactic antibiotic administration could not be evaluated in the present study. Sixth, we were not able to perform complete hospital‐level adjustments for each specific antibiotic of broad‐spectrum antibiotic, which might have resulted in residual confounding related to institutional prescribing practices.

In conclusion, the use of third‐ or fourth‐generation cephalosporins and piperacillin–tazobactam as antimicrobial prophylaxis was associated with a lower incidence of postoperative intra‐abdominal infections compared with first‐ or second‐generation cephalosporins in patients undergoing PD, irrespective of the presence of a preoperative biliary stent. Broad‐spectrum antibiotics may be suitable for perioperative antibiotic prophylactic in PD.

## Author Contributions

**Hiroki Kitagawa:** conceptualization, methodology, investigation, writing – original draft, formal analysis, visualization. **Shotaro Aso:** methodology, data curation, investigation, validation, formal analysis, supervision, visualization, writing – review and editing, software, resources. **Yuki Hirano:** methodology, data curation, investigation, formal analysis, writing – review and editing, supervision, project administration. **Kenichiro Uemura:** writing – review and editing. **Keita Kouzu:** writing – review and editing. **Satoru Matsuda:** writing – review and editing. **Hiroki Matsui:** writing – review and editing, data curation, resources. **Kiyohide Fushimi:** writing – review and editing, data curation, resources. **Junichi Sasaki:** writing – review and editing, project administration. **Yuko Kitagawa:** writing – review and editing. **Hideo Yasunaga:** writing – review and editing, supervision, funding acquisition, resources.

## Funding

This work was supported by grants from the Ministry of Health, Labour and Welfare, Japan (23AA2003 and 24AA2006).

## Ethics Statement

This study was approved by the Institutional Review Board of the University of Tokyo [approval number: 3501‐(5)]. The requirement for informed consent was waived because of the anonymous nature of the data.

## Conflicts of Interest

Dr. Yuko Kitagawa is Editor‐in‐Chief of Annals of Gastroenterological Surgery. Dr. Satoru Matsuda is member of editorial board of Annals of Gastroenterological Surgery. There are no other conflicts of interest to disclose.

## Supporting information


**Table S1.** ICD‐10 codes and procedures used to define each postoperative complication.

## Data Availability

Because the data in this study was extracted from a nationwide database, data use requires approval of the Ministry of Health, Labour, and Welfare, Japan.
