# Supplementary material for: Effect of Broad‐Spectrum Antibiotic Prophylaxis on Post‐Pancreatoduodenectomy Infectious Complications: Nationwide Inpatient Database Study in Japan
Source: Ann Gastroenterol Surg. 2026 Feb 26;10(4):1231–8. doi: 10.1002/ags3.70198 (PMC13327081; doi:10.1002/ags3.70198)
Supplement: Supplementary file 1 — Table S1. ICD‐10 codes and procedures used to define each postoperative complication. [file AGS3-10-1231-s001.docx]

**Supplemental Table 1. ICD-10 codes and procedures used to define each postoperative complication**

|  | ICD-10 codes | Procedures (searched in the original Japanese codes) |
| --- | --- | --- |
| Intra-abdominal infections | K65, T813, T814, K833, K868, K918 | Procedures for managing bile leakage, anastomotic leakage, and intra-abdominal abscess, or long-term drainage tube placement (defined as the insertion of a drainage tube for ≥ 3 weeks after surgery). |
| Postoperative pancreatic fistula | K868, K918 |  |
| Bile leakage | K833 |  |
| *Clostridioides difficile* infection | A47 |  |
| Post-pancreatectomy hemorrhage |  | Procedures for transcatheter arterial embolization |

ICD-10, International Classification of Diseases, Tenth Revision
